# A Comparative Analysis of Video-Assisted Thoracoscopic Surgery and Thoracotomy in Non-Small-Cell Lung Cancer in Terms of Their Oncological Efficacy in Resection: A Systematic Review

**DOI:** 10.7759/cureus.25443

**Published:** 2022-05-29

**Authors:** Tuheen S Nath, Nida Mohamed, Paramjot K Gill, Safeera Khan

**Affiliations:** 1 Surgical Oncology, California Institute of Behavioral Neurosciences & Psychology, Fairfield, USA; 2 Surgical Oncology, Tata Medical Centre, Kolkata, IND; 3 Trauma and Acute Care Surgery, California Institute of Behavioral Neurosciences & Psychology, Fairfield, USA; 4 Obstetrics and Gynaecology, California Institute of Behavioral Neurosciences & Psychology, Fairfield, USA; 5 Health Leadership, Royal Roads University, Victoria, CAN; 6 General Practice, Dashmesh Hospital, Ropar, IND; 7 Internal Medicine, California Institute of Behavioral Neurosciences & Psychology, Fairfield, USA

**Keywords:** lymph node dissection, long-term outcomes, oncological outcomes, lung cancer, non-small cell lung cancer, open lobectomy, open thoracotomy, vats, video-assisted thoracoscopic surgery

## Abstract

Video-assisted thoracoscopic surgery (VATS) is considered the standard procedure for surgical resection in non-small-cell lung cancer (NSCLC). However, there is still lingering speculation on its adequacy of lymph node (LN) dissection or sampling and the long-term survival benefits when compared to open thoracotomy. Given the above, we conducted a systematic review comparing VATS and thoracotomy in terms of their oncological effectiveness in resection.

We explored major research literature databases and search engines such as MEDLINE, PubMed, PubMed Central, Google Scholar, and ResearchGate to find pertinent articles. After the meticulous screening, quality check, and applying relevant filters according to our eligibility criteria, we identified 16 studies relevant to our research question, out of which one was a randomized controlled trial, one meta-analysis, and 14 were observational studies. The study comprised 44,673 patients with NSCLC, out of whom 15,093 patients were operated by VATS and the remaining 29,580 patients by thoracotomy. The results indicate that VATS is equivalent to thoracotomy in total LNs (N1 + N2) and LN stations dissected. However, a thoracotomy may achieve slightly better mediastinal lymph node dissection (N2) in terms of assessing a greater number of mediastinal lymph nodes and nodal stations. This may be attributed to a better visual field during mediastinal nodal clearance by an open approach. Also, nodal upstaging was consistently more common with an open approach. In terms of long-term outcomes, both overall survival and disease-free survival rates were similar between the two groups, with VATS offering a slightly better survival benefit.

Irrespective of the increased rates of nodal upstaging by an open approach, we conclude that VATS should be considered a highly efficient alternative to thoracotomy in both early and locally advanced NSCLC.

## Introduction and background

Lung cancer is the most prevalent type of cancer in both sexes and accounts for 18.4% of all cancer-related mortality globally [1]. Non-small-cell lung cancer (NSCLC) is by far the most common type comprising a majority of all lung cancer diagnoses. The treatment modality for early-stage NSCLC consists of surgical resection and lymph node (LN) dissection or sampling [2,3]. Historically, surgical intervention has involved open approaches, including open lobectomy, segmentectomy, or wedge resection. However, these rib-spreading invasive approaches are associated with many post-operative morbidities [4].

In this prelude, minimally invasive surgical techniques such as video-assisted thoracoscopic surgery (VATS) are being readily used nowadays to reduce surgical trauma associated with open procedures. The first documented evidence of VATS for lung cancer resection and mediastinal lymph node dissection (MLND) was provided by McKenna in 1994 [5]. Since its inception, there has been a gradual and widespread adoption of VATS as the standard operative procedure for early-stage NSCLC [6,7]. Many studies are delineating the efficacy of VATS over open thoracotomy concerning reduced post-operative pain, improved post-operative pulmonary function, shorter chest tube duration, shorter hospital stay as well as decreased incidence of other post-operative morbidities, including supraventricular arrhythmias, myocardial infarction, pulmonary embolism, and empyema [6,8-10].

However, despite its growing popularity and well-documented improved short-term outcomes, a nagging concern remains regarding the efficacy of VATS to achieve equivalent oncological resection due to its minimally invasive approach compared to open procedures. The oncological efficacy of resection is generally measured by the adequacy and extent of LN dissection or staging and the long-term survival rates. According to the National Comprehensive Cancer Network (NCCN) guidelines, sampling of a minimum of three N2 stations or complete mediastinal nodal dissection is recommended [11]. In a study conducted by Wang et al. in patients with clinical N0/1 (cN0/1) and pathological N2 (pN2) NSCLC, there was a significant difference in the overall survival (OS) and five-year disease-free survival (DFS) rates in favor of the group fulfilling the NCCN criteria for mediastinal lymphadenectomy versus the other (72% ± 2% vs. 63% ± 4% [OS] and 58% ± 2% vs. 49% ± 4% [DFS]) [12].

The previous literature on lung cancer surgery suggests a lack of consistency in lymph node assessment with VATS compared to thoracotomy. Several studies have shown an improved lymph node dissection and increased nodal upstaging from cN0/1-pN1/2 with thoracotomy compared to thoracoscopic approaches [13-16]. Incomplete lymph node dissection and staging may have a detrimental effect on the long-term outcome of surgery by leaving behind occult cancer. This raises a query regarding the optimal surgical approach to achieve ideal oncological resection and long-term outcomes in patients with NSCLC.

Given the above, we conducted a systematic review to examine the differences in the efficacy of lymph node dissection and long-term survival between VATS and open thoracotomy in patients with NSCLC.

## Review

Methods

We used the Preferred Reporting Items for Systematic Reviews and Meta-Analyses (PRISMA) guidelines and principles to design this systematic review and report the results [17].

Search Strategy

We used major research literature databases and search engines such as MEDLINE, PubMed, PubMed Central (PMC), Google Scholar, and ResearchGate to search appropriate keywords and Medical Subject Headings (MeSH) thesaurus and find relevant articles pertaining to the topic [18-21].

The final combined MeSH strategy for PubMed, PMC and MEDLINE is as follows: Non-small cell Lung Cancer OR Lung Cancer OR Squamous Cell Carcinoma Lung OR Adenocarcinoma Lung OR Large Cell Carcinoma Lung OR NSCLC OR ("Carcinoma, Non-Small-Cell Lung/analysis"[Majr] OR "Carcinoma, Non-Small-Cell Lung/mortality"[Majr] OR "Carcinoma, Non-Small-Cell Lung/surgery"[Majr] OR "Carcinoma, Non-Small-Cell Lung/therapy"[Majr]) AND VATS OR Video-Assisted Thoracoscopic Surgery OR ("Thoracic Surgery, Video-Assisted/adverse effects"[Majr] OR "Thoracic Surgery, Video-Assisted/mortality"[Majr] OR "Thoracic Surgery, Video-Assisted/statistics and numerical data"[Majr] OR "Thoracic Surgery, Video-Assisted/therapeutic use"[Majr] OR "Thoracic Surgery, Video-Assisted/therapy"[Majr]) AND Open Lobectomy OR Open Thoracotomy OR Open Segmentectomy OR ("Thoracotomy/adverse effects"[Majr] OR "Thoracotomy/mortality"[Majr] OR "Thoracotomy/statistics and numerical data"[Majr] OR "Thoracotomy/therapeutic use"[Majr] OR "Thoracotomy/therapy"[Majr]).

The keywords used for search in Google Scholar and ResearchGate include "Video-assisted Thoracoscopic Surgery", "VATS", "Open Thoracotomy," "Open Lobectomy, "Non-Small Cell Lung Cancer", "Lung Cancer", "Oncological Outcomes", "Long-term Outcomes", "Lymph Node Dissection". To find relevant articles, these keywords were combined in varying combinations using Booleans "AND", "OR", "NOT".

Inclusion and Exclusion Criteria

We included observational studies, randomized controlled trials (RCTs) and review articles published in the English language in the last 10 years, focusing on the adult and geriatric population (>18 years), and relevant to our research question. We excluded articles focusing on the pediatric population (<18 years), case reports, letters, expert opinions, animal studies, and unpublished or grey literature.

A detailed description of the inclusion and exclusion criteria is given in Table 1.

**Table 1:** Detailed inclusion and exclusion criteria

Inclusion criteria	Exclusion criteria
1. Papers from the past 10 years	1. Papers before the past 10 years
2. Papers published in the English language	2. Papers not published in the English language
3. Papers focusing on adult and geriatric population (>18 years)	3. Papers discussing pediatric population (<18 years)
4. Non-small-cell lung cancer	4. Small-cell lung carcinoma, metastasis
5. Lobectomy, segmentectomy, wedge resection	5. Pneumonectomy, metastatectomy
6. Video-assisted thoracoscopic surgery, open thoracotomy	6. Robotic-assisted thoracoscopic surgery
7. Observational studies, randomized controlled trials, reviews and meta-analyses	7. Case reports, letters, expert opinions, animal studies, grey literature, unpublished literature
8. Papers relevant to the question	8. Papers irrelevant to the question

Analysis of Study Quality/Bias

We critically evaluated 17 selected studies for quality, using standardized quality assessment tools, and 16 studies qualified as medium or high quality, which were included in the review. The following tools were used: (1) for observational studies, Newcastle-Ottawa scale; (2) for systematic reviews and meta-analyses, Assessment of Multiple Systematic Reviews (AMSTAR) tool; (3) for traditional reviews, Scale for the Assessment of Narrative Review Articles (SANRA) checklist; (4) for RCTs, Cochrane risk-of-bias assessment tool.

The detailed overall scores and quality for each study are provided in Tables 2, 3.

**Table 2:** Summary of the Newcastle-Ottawa risk-of-bias tool for observational studies N/A, not applicable Quality check was done as per the Newcastle-Ottawa Scale (1, 0, N/A).

Selection	Medbery et al. (2016) [22]	Ramos et al. (2012) [23]	Stephens et al. (2014) [10]	Zhong et al. (2013) [24]	Chen et al. (2017) [25]	Witte et al. (2015) [26]	Paul et al. (2014) [27]	Higuchi et al. (2014) [28]	D’Amico et al. (2011) [29]	Hanna et al. (2013) [30]	Merritt et al. (2013) [13]	Boffa et al. (2012) [15]	Lee et al. (2013) [16]	Liu et al. (2015) [31]
Representativeness of the exposed cohort	1	1	1	1	1	1	1	1	1	1	1	1	1	1
Selection of the non-exposed cohort	1	1	1	1	1	1	1	1	1	1	1	1	1	1
Ascertainment of exposure	1	1	1	1	1	1	1	1	1	1	1	1	1	1
Demonstration that outcome of interest was not present at the start of the study	1	1	1	1	1	1	1	1	1	1	1	1	1	1
Comparability														
Study controls for most important factor (age)	1	1	1	1	1	1	1	1	1	1	1	1	1	1
Study controls for any additional factor(s)	1	1	1	1	1	1	1	0	1	1	1	1	1	1
Outcome														
Assessment of outcome	1	1	1	1	1	1	1	1	1	1	1	1	1	1
Was follow-up long enough for outcomes to occur?	0	1	1	0	1	1	1	1	1	1	1	N/A	1	1
Adequacy of follow-up of cohorts	0	0	1	0	0	0	1	0	0	0	0	N/A	0	0
Total	7/9	8/9	9/9	7/9	8/9	8/9	9/9	7/9	8/9	8/9	8/9	7/9	8/9	8/9
Quality	Medium	High	High	Medium	High	High	High	Medium	High	High	High	Medium	High	High

**Table 3:** Summary of the AMSTAR tool for systematic reviews and meta-analyses AMSTAR, Assessment of Multiple Systematic Reviews; RoB, risk of bias; PICO, patient/population, intervention, comparison, outcome

AMSTAR criteria (yes, partial yes, no)	Study
	Zhang et al. (2016) [32]
Did the research questions and inclusion criteria for the review include PICO components?	Yes
Did the report of the review contain an explicit statement that the review methods were established prior to the conduct of the review and did the report justify any significant deviations from the protocol?	Partial yes
Did the review authors explain their selection of the study designs for inclusion in the review?	Yes
Did the review authors use a comprehensive literature search strategy?	Partial yes
Did the review authors perform study selection in duplicate?	Yes
Did the review authors perform data extraction in duplicate?	Yes
Did the review authors provide a list of excluded studies and justify the exclusions?	Yes
Did the review authors describe the included studies in adequate detail?	Partial yes
Did the review authors use a satisfactory technique for assessing the RoB in individual studies that were included in the review?	Yes
Did the review authors report on the funding sources for the studies included in the review?	No
If meta-analysis was performed, did the review authors use appropriate methods for statistical combination of results?	Partial yes
If meta-analysis was performed, did the review authors assess the potential impact of RoB in individual studies on the results of the meta-analysis or other evidence synthesis?	Yes
Did the review authors account for RoB in individual studies when interpreting/discussing the results of the review?	Yes
Did the review authors provide a satisfactory explanation for, and discussion of, any heterogeneity observed in the results of the review?	Yes
Did the review authors provide a satisfactory explanation for, and discussion of, any heterogeneity observed in the results of the review?	Yes
Did the review authors report any potential sources of conflict of interest, including any funding did they receive for conducting the review?	Yes
Total score	13/16 (high quality)

Data Extraction

Two investigators independently extracted data from the eligible studies and examined them for the following: (1) type of study, (2) number of participants, (3) number of LNs and LN stations dissected, (4) rates of nodal upstaging and (5) long-term outcomes.

Results

**Figure 1:**
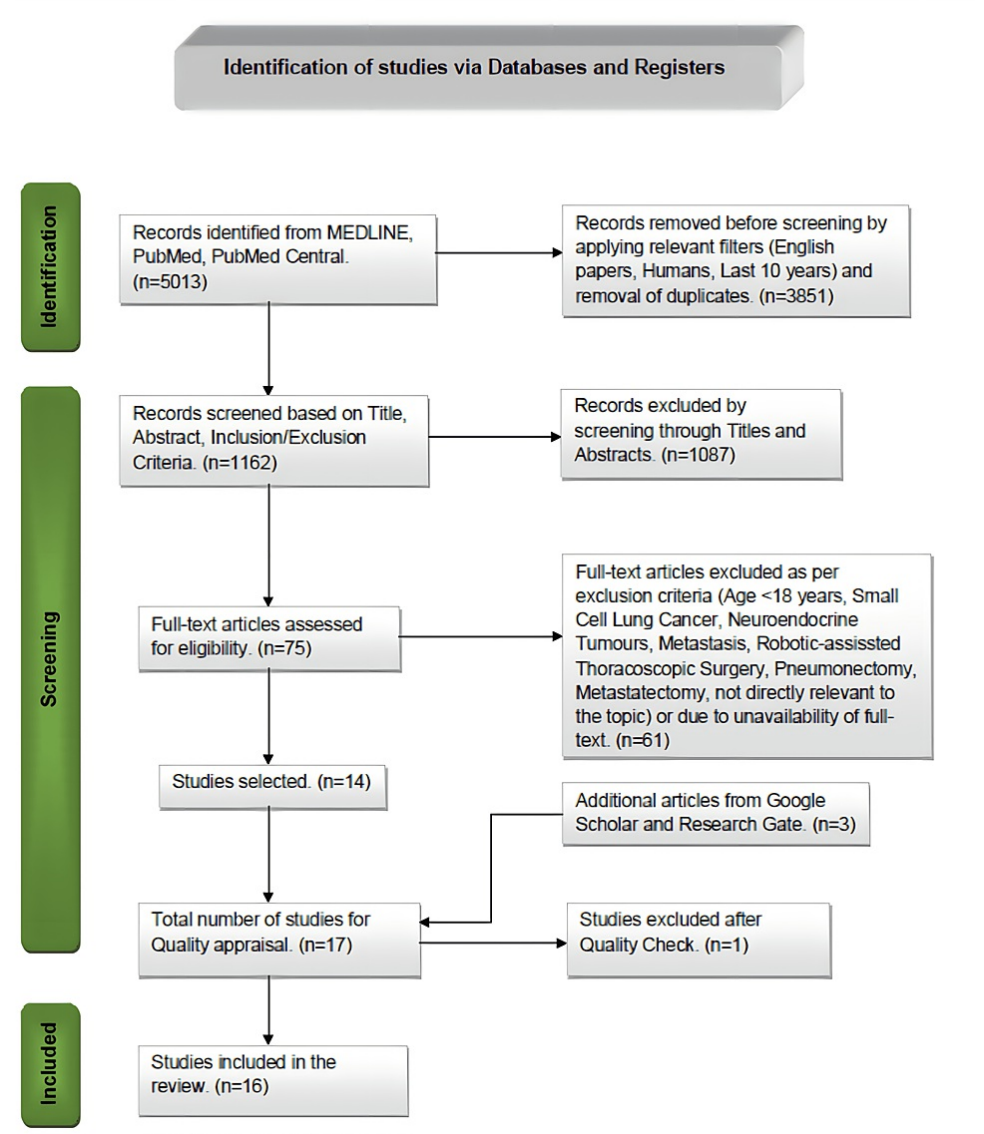
Preferred Reporting Items for Systematic Reviews and Meta-Analyses (PRISMA) flow diagram

A total of 5013 articles were identified in our initial search of MEDLINE, PubMed, and PMC databases. Out of them, 3851 articles were discarded after applying relevant filters as per our eligibility criteria (last 10 years, human studies, English papers) and duplicates were removed. Two individual investigators then screened the remaining articles (n=1162) based on titles, abstracts, full-text, and detailed inclusion-exclusion criteria. After the meticulous screening, we were left with 14 articles about our research question. Additional three articles were added by searching the relevant keywords in Google Scholar and ResearchGate directly relevant to our topic. A total of 17 studies were included for a thorough quality/bias assessment using standardized quality assessment tools. One study was excluded after quality appraisal, and the final 16 studies were included in this systematic review. The PRISMA 2020 flow diagram is depicted in Figure 1 [17].

Of the 16 included studies, there were 14 observational studies, one RCT and one meta-analysis. They included 44,673 patients with NSCLC, out of whom 15,093 patients underwent VATS, and 29,580 patients underwent thoracotomy. We identified 14 studies that compared the efficacy of lymph node dissection and rates of nodal upstaging following VATS or thoracotomy. They included 44,413 patients, of whom 14,923 belonged to the VATS group, and 29,490 belonged to the thoracotomy group. There was no significant difference in the total number of lymph nodes (N1 + N2) and lymph node stations resected between the VATS and thoracotomy groups. However, a thoracotomy may be slightly better than VATS in terms of MLND (N2) alone. Nodal upstaging (N1/N2) was also moderately more common with an open approach.

In another sub-group, we analysed the long-term outcomes of surgery by comparing the three-year and five-year OS and DFS rates between VATS and thoracotomy. We identified 10 studies comparing the two groups' five-year OS, five-year DFS, three-year OS, and three-year DFS. They included 9164 patients, of whom 2674 patients underwent VATS and 6490 patients underwent open procedures. OS and DFS were nearly similar between the two groups, with VATS conferring a slightly better survival benefit.

Discussion

We analysed the oncological efficacy of resection conferred by VATS and open thoracotomy by comparing the completeness of lymph node dissection or sampling and the survival rates in patients with NSCLC.

In patients with lung cancer, a pre-operative staging routinely done by computed tomography (CT) scan, positron emission tomography (PET) scan or mediastinoscopy may not always be accurate. This is evident by post-operative nodal upstaging after lobectomy with systematic lymph node dissection. In a prospective trial including 502 clinical Stage I NSCLC patients, who underwent surgical resection and complete lymph node dissection, D'Cunha et al. demonstrated that 38.3% were upstaged post-operatively or had inaccuracies in diagnosis [33]. An extensive and complete lymph node assessment is vital for lung cancer surgery. It helps in accurate post-operative staging and minimize the chance of leaving behind occult malignancy. This, in turn, has implications in guiding adjuvant chemotherapy, if required, which has proven beneficial in upstaged NSCLC patients [34,35].

Also, the number of dissected lymph nodes directly correlates with the OS. In a retrospective study conducted by Jeon et al. including 211 patients with clinical Stage I NSCLC, patients were divided into two groups. One group underwent lobectomy with complete mediastinal lymph node dissection (14.09 ± 7.57 LNs), and the other underwent lobectomy with selective lymph node sampling (7.50 ± 5.44 LNs). The results favored the group with greater resected lymph nodes in overall survival [36]. Ou et al. also demonstrated a definite survival benefit in patients with more dissected lymph nodes [37]. A recent large retrospective study by David et al. reported that the number of dissected lymph nodes served as a significant positive prognostic factor [38]. The current NCCN guidelines also vouch for a minimum of three N2 nodal station dissections or sampling.

Although the short-term benefits of VATS lobectomy are well documented, the efficacy of lymph node dissection and subsequent long-term outcomes is questionable when compared to thoracotomy lobectomy. With this in mind, we selected 16 studies for our systematic review that compared VATS and open thoracotomy for the effectiveness of nodal dissection and overall and disease-free survival.

Comparison of Efficacy of Lymph Node Dissection

Fourteen of our included studies compared the effectiveness of nodal dissection achieved by VATS versus thoracotomy. They included 12 retrospective cohort studies, one RCT and one meta-analysis. They all assessed the number of lymph nodes and stations dissected and rates of nodal upstaging. Still, they differed regarding study design, sample size, geographic and demographic aspects of the participants, and certain eligibility criteria.

In a retrospective study conducted by Ramos et al., 296 patients with clinical Stage I-II NSCLC from a single institution in France were enrolled [23]. Out of them, 96 surgical resections and LND were via VATS and the remaining 200 via posterolateral thoracotomy. Pre-operative assessments for both the groups were the same, and there was comparability between them concerning age, gender, and existing comorbidities except cardiopathy. The study showed that VATS achieved a better total (5.1 ± 1.1 vs. 4.5 ± 1.2) as well as mediastinal (3.4 ± 0.9 vs. 3.2 ± 0.9) nodal station dissection. The two procedures were similar in MLND (17.7 ± 8.2 vs. 18.2 ± 9.3). However, thoracotomy was slightly superior in harvesting total LNs (N1 + N2; 22.6 ± 9.4 vs. 25.4 ± 10.8).

Stephens et al. conducted a retrospective study on 963 patients with clinical Stage I NSCLC from Texas (VATS, n=307; posterolateral thoracotomy, n=656) [10]. Pre- and post-operative staging were done in accordance with the American Joint Committee on Cancer (AJCC) guidelines [39]. The results showed that the two procedures were similar in the number of LN stations assessed in propensity-matched analysis (4.2 vs. 4.3). However, VATS was slightly better in assessing hilar and peribronchial stations (2.5 vs. 1.6). There was no difference in N2 station sampling (2.6 vs. 2.5). In terms of post-operative nodal upstaging, the two groups had similar rates (35 % VATS vs. 38% open).

A study carried out by Zhong et al. including 157 Chinese patients with NSCLC showed that VATS achieved identical results in terms of total LND (17.4 ± 6.1 vs. 18.1 ± 7.2) as well as MLND (11.7 ± 5.6 vs. 12 ± 5.1) when compared to thoracotomy [24]. Total LN stations and MLN stations sampled were also similar. However, this study was limited by a small sample size.

D'Amico et al. also reported results similar to Zhong et al.'s in terms of total (N1 + N2) nodes (median, 4) and MLN removed [29]. This study was carried out retrospectively and included 388 patients with NSCLC who were registered on the NCCN NSCLC database (VATS, n=199; open, n=189). Patients who had at least three MLN assessments were included in the study to comply with NCCN guidelines. Although the efficacy of LN dissection was similar between the groups, rates of nodal upstaging were slightly higher in the open group than VATS (8.8% vs. 14.5%). This may be partly attributed to selection bias as more patients with clinical N1 disease were operated on by VATS. Also, we must consider that it was a multi-institutional study with a varied spectrum of clinical practice analysed.

A more recent large, national-level study by Medbery et al. based on the National Cancer Database (NCDB), followed up by the American Cancer Society and American College of Surgeons, sought to compare rates of nodal upstaging following VATS (n=4935) and thoracotomy (n=12,048) [22]. The results showed that VATS harvested a greater total number of LNs (≥9 LNs, 43.7% vs. 38.8%) in the unmatched analysis as well as in the propensity-matched analysis of 4437 patients (mean, 10.3 vs. 9.7 LNs). Despite VATS achieving better nodal dissection, rates of nodal upstaging were slightly more common following the open procedure (11.9 % vs. 10.1%). Among them, upstaging from cN0-pN1 was more frequently observed with thoracotomy (8% vs. 6.9%). This may be due to information bias, as non-uniformity of pre-operative staging methods within the large cohort might have had implications for the clinical staging.

In contrast to the findings of Medbery et al., a systematic review and meta-analysis conducted the same year by Zhang et al. extrapolated that a lesser number of total LNs were assessed by VATS when compared to thoracotomy (95% CI -0.28 to 0.06, p=0.20) [32]. This discrepancy was mainly related to inadequate MLND by VATS (95% CI -1.38 to 0.49, p<0.0001).

In a retrospective study by Merritt et al., 129 patients with clinical N0 lung cancer at Standford University Hospital who underwent surgical resection either by VATS (n=60) or open lobectomy (n=69) were evaluated for efficacy of LN assessment [13]. The results showed that open lobectomy achieved considerably better dissection of a total number of LNs (14.7 ± 1.3 vs. 9.9 ± 0.84). This may be attributed to a much greater number of N2 nodes harvested by thoracotomy (8.5 ± 1.0 vs. 4.7 ± 0.55), which supports findings of the previous study. There was not much difference in the adequacy of N1 nodal assessment (6.2 ± 0.5 vs. 5.2 ± 0.5). The rates of nodal upstaging from cN0-pN1/2 were also much greater with thoracotomy (24.6% vs. 10%). This may be due to more T1 carcinomas (68.3% vs. 52.2%) and fewer T2, T3 cancers in the VATS group than open. The fewer LNs harvested in VATS may also be partly due to one of the surgeons opting for systematic nodal sampling instead of complete lymphadenectomy for VATS.

A large study based on the Society of Thoracic Surgeons - General Thoracic Database (STS-GTD) conducted by Boffa et al. comprised 11,531 pulmonary resections (VATS, n=4394; open, n=7137) [15]. The results showed that thoracotomy was associated with increased rates of nodal upstaging as compared to VATS (14.3% vs. 11.6%). This was mainly due to VATS inadequate hilar or peribronchial nodal assessment, as evident by the cN0-pN1 upstaging rates (9.3 vs. 6.7), which contradicts the findings of Stephens and colleagues. MLN assessment was similar between the two cohorts, which contradicts the findings of Merritt et al. and Zhang et al. This increased N1 upstaging by thoracotomy may be explained by a possible increase in clinical N0 staging. The STS-GTD did not have any mention of staging methodology. Also, there might have been information bias as the database did not contain information on tumor location. Central lesions are more prone to have unknown nodal metastasis than peripheral tumors [40,41]. Selection bias in favorably selecting VATS for peripheral T1 tumors may have led to low upstaging rates. Lastly, a surgeon's preferred practice and experience also influenced the results; while comparing 989 VATS cases from 18 VATS-preferring institutions/surgeons with 3668 open cases from thoracotomy-preferring case-submitters, N1 upstaging rates were found to be similar (8.7%).

Lee and colleagues reported results similar to those by Zhang et al. and Merritt et al. [16]. They concluded that thoracotomy is better than VATS in terms of LN evaluation (total LN dissected: VATS 11.3 vs. open 14.3). MLND achieved by thoracotomy was much more efficient (8.5 ± 7.1 vs. 5.7 ± 4.3). N1 dissection was similar, which contradicts the previous study. Nodal upstaging from cN1-pN2/3 was also more common with open approaches (23% vs. 13.8%).

**Figure 2:**
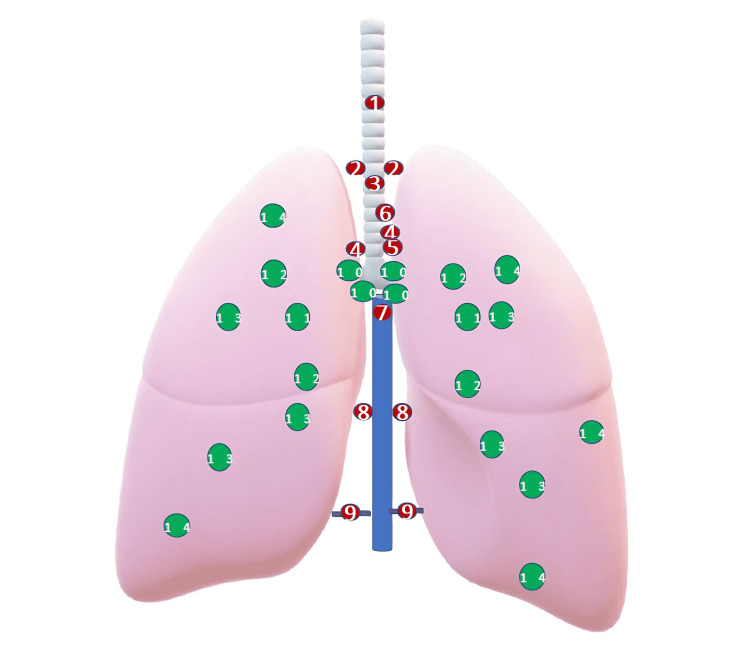
N1 and N2 lymph node mapping and nodal zones as per International Association for the Study of Lung Cancer Shown in red - N2 (mediastinal lymph node stations): 1, highest mediastinal; 2, paratracheal; 3, pretracheal (anterior mediastinal); 4, tracheobronchial; 5, subaortic (Botallo’s); 6, paraaortic (ascending aortic); 7, subcarinal; 8, paraesophageal; 9, pulmonary ligament. Shown in green - N1 (hilar and peribronchial node stations): 10, hilar; 11, interlobar; 12, lobar; 13, segmental; 14, subsegmental. The figure is adapted from Refs [43, 44] and was created by the first author.

The only prospective RCT included in the review conducted by Palade and colleagues analysed 64 German patients with clinical Stage I NSCLC. They were operated by either VATS (n=32) or open lobectomy (n=32) [42]. The results showed that VATS was equally effective as thoracotomy in terms of overall nodes dissected (right Side, 24 ± 7.5 vs. 25.2 ± 7.2; left side, 25.1 ± 9.3 vs. 21.1 ± 10.4). Lymph nodes were dissected according to zones classified by the International Association for the Study of Lung Cancer (IASLC) [43]. There was no significant difference in any of the zonal dissection also. However, this RCT was limited by the small number of its participants. This finding was supported by a Canadian retrospective study conducted by Hanna et al. in the same year including a larger cohort size of 608 NSCLC patients [30]. The study showed no statistical difference between VATS and thoracotomy in nodal sampling at different stations. The IASLC nodal mapping is depicted below in Figure 2.

In 2014, Liu et al. reported no difference in the number of total LNs harvested (VATS, 22-36 vs. open, 22-40) and MLNs dissected (12-23 vs. 12-28) between VATS (n=123) and thoracotomy (n=89) [31]. The same year, Paul et al. retrospectively analysed the SEER-Medicare database, a large US-based national database [27]. The study included 6008 patients with NSCLC resected surgically either by thoracotomy (n=4715) or VATS (n=1293). They found that VATS resected more LNs in both matched (19.9 vs. 17.6) and unmatched analysis (20.1 vs. 17.7). However, the findings may have been biased because it was a multi-institutional study with varied surgeon experience and expertise.

Finally, we also included a study conducted by Chen et al. that focused on 411 patients with locally advanced NSCLC (Stages II, III), who underwent surgical resection and LN dissection by either VATS (n=250) or thoracotomy (n=161) [25]. Both procedures harvested similar number of total LNs (15.6 ± 9.2 vs. 14.7 ± 7.9), hilar and peribronchial nodes (5.3 ± 4 vs. 5.3 ± 3.7) and mediastinal nodes (10.3 ± 7 vs. 9.4 ± 5.8). Interestingly, the number of LNs resected by VATS improved with more operative time.

On interpreting and analysing the studies mentioned above, we found that despite VATS offering a more or less equivalent nodal dissection, nodal upstaging was consistently more common with thoracotomy. This may be explained by the open approach's more targeted and meticulous clearance of suspected LNs, owing to the better visual field. This may also have been affected by facility type. Also, as most of our included studies were observational studies, an inherent selection bias might have led to larger tumors with a greater tendency of a positive nodal burden being operated openly.

The brief descriptions of each study, including the year of publication, author name, number of patients, type of study, results, and conclusions of the authors regarding LN assessment/dissection, are listed in Table 4.

**Table 4:** Synopsis of articles comparing VATS and thoracotomy in terms of number of LNs harvested, number of LN stations resected and nodal upstaging VATS, video-assisted thoracoscopic surgery; LN, lymph node; MLND, mediastinal lymph node dissection; c, clinical stage; p, pathological stage

Author and year of publication	Interventions studied	Number of patients	Type of study	Result	Conclusion
Medbery et al., 2016 [22]	VATS vs. thoracotomy	16,983; VATS (n=4935), thoracotomy (n=12,048)	Retrospective cohort	VATS was associated with a greater number of regional lymph nodes examined (mean 10.3 vs. 9.7 in propensity-matched groups). Nodal upstaging VATS vs. open, 10.3% vs. 12.8%; unmatched analysis, 10.1% vs. 11.9%; propensity-matched analysis (N1; 6.9% vs. 8%).	VATS provided better lymph node evaluation as compared to open lobectomy. However, nodal upstaging (N1) was more common for thoracotomy than VATS.
Palade et al., 2013 [42]	VATS vs. open lobectomy	64; VATS (n=32), open lobectomy (n=32)	Randomized control trial	VATS vs. open, right-sided LN dissected (24 vs. 25.2), left-sided LN dissected (25.1 vs. 21.1). There was no significant difference concerning lymph nodes dissected from each zone between the two.	VATS was equally effective as open lobectomy concerning MLND.
Ramos et al., 2012 [23]	VATS vs. posterolateral thoracotomy	296; VATS (n=96), posterolateral thoracotomy (n=200)	Retrospective cohort	VATS vs. open, MLND (17.7 ± 8.2 vs. 18.2 ± 9.3), total mean lymph nodes dissected (22.6 ± 9.4 vs. 25.4 ± 10.8); nodal upstaging c-I/II to p-IIIA (VATS, 5.4% vs. open, 9%).	There was no difference between VATS and posterolateral thoracotomy in terms of MLND. However, thoracotomy was slightly more effective for overall (mediastinal + lobar) LN assessment. They were statistically similar in terms of nodal upstaging (p>0.05).
Stephens et al., 2014 [10]	VATS vs. open lobectomy	963; VATS (n=307) open lobectomy (n=656)	Retrospective cohort	VATS vs. open, LN stations sampled (4.2 vs. 4.3); nodal upstaging (VATS, 35% vs. open, 38%)	VATS was equally effective as open lobectomy in sampling different LN stations. Nodal upstaging rates were also similar.
Zhong et al., 2013 [24]	VATS vs. open lobectomy	157; VATS (n=67), open lobectomy (n=90)	Retrospective cohort	VATS vs. open, total LN removed (17.4 ± 6.1 vs. 18.1 ± 7.2), MLND (11.7 ± 5.6 vs. 12 ± 5.1), total LN stations removed (7.6 ± 1.9 vs 7.8 ± 2.3), MLN stations removed (4.5 ± 1.1 vs. 4.7 ± 1.3).	There was no difference between VATS and open lobectomy in terms of the efficacy of LN dissection.
Chen et al., 2016 [25]	VATS vs. thoracotomy	411; VATS (n=250), thoracotomy (n=161)	Retrospective cohort	VATS vs thoracotomy, total LN removed (15.6 ± 9.2 vs. 14.7 ± 7.9), total LN stations resected (5.5 ± 1.8 vs. 5.5 ± 1.6).	There was no significant difference in LN resection between the two approaches.
Paul et al., 2014 [27]	VATS vs. thoracotomy	6008; VATS (n=1293), open (n=4715)	Retrospective cohort	VATS vs. thoracotomy, total LN removed in the full group (mean 20.1 vs. 17.7), total LN removed in the matched group (mean 19.9 vs. 17.6).	VATS approach did not compromise the efficacy of LN dissection.
D'Amico et al., 2011 [29]	VATS vs. thoracotomy	388; VATS (n=199); open (n=189)	Retrospective cohort	Total number of N1-N2 nodes removed for each group (median, 4), p=0.06, nodal upstaging cN0- pN1/2 (VATS, 8.8% vs. open, 14.5%).	There was no difference in surgical LN resection between the two procedures. However, upstaging was more common in the thoracotomy group.
Merritt et al., 2013 [13]	VATS vs. open lobectomy	129; VATS (n=60), open (n=69)	Retrospective cohort	VATS vs. open lobectomy - total number of nodes dissected (9.9 ± 0.8 vs. 14.7 ± 1.3), MLND (4.7 ± 0.55 vs. 8.5 ± 1), nodal upstaging cN0-pN1/2 (VATS, 10% vs. open, 24.6%).	Open lobectomy was superior to VATS in terms of LN dissection. Nodal upstaging was also much more frequent with open procedures.
Boffa et al., 2012 [15]	VATS vs. thoracotomy	11,531; VATS (n=4394), open (n=7137)	Retrospective cohort	Nodal upstaging - VATS vs thoracotomy: overall (11.6% vs. 14.3%), cN0-pN1 (6.7% vs. 9.3%), CN0-pN2 (4.9% vs. 5.0%), propensity-matched cN0-pN1 (6.8% vs. 9%).	N1 upstaging was less common with VATS than open procedures, indicating the thoracoscopic approach's inadequate peribronchial and hilar nodal evaluation.
Lee et al., 2013 [16]	VATS vs. thoracotomy	416; VATS (n=208), thoracotomy (n=208)	Retrospective cohort	VATS vs. thoracotomy - total LN removed (11.3 vs. 14.3), N1 nodes (6.3 ± 4.4 vs. 6.5 ± 3.3), N2 nodes (5.7 ± 4.3 vs. 8.5 ± 7.1), total LN stations evaluated (3.1 vs. 3.8), nodal upstaging cN1-pN2/3 (VATS, 13.8% vs. open 23%).	Thoracotomy was better than VATS in terms of LN evaluation. This is mainly because of VATS' inadequate N2 resection (MLND). Upstaging was also more common with thoracotomy.
Hanna et al., 2013 [30]	VATS vs. open lobectomy	608; VATS (n=196), open (n=412)	Retrospective cohort	No significant statistical difference was noted in LN sampling at different stations between the two procedures. Nodal upstaging cN0-pN1 (VATS, 5.8% vs. 7.4%).	There was no difference in LN sampling between the two procedures.
Liu et al., 2014 [31]	VATS vs. thoracotomy	212; VATS (n=123), open (n=89)	Retrospective cohort	VATS vs. thoracotomy - total LN resected (22-36 vs. 22-40, p=0.164), MLND (12-23 vs. 12-28, p=0.110); total LN stations harvested were the same.	There was no difference in surgical LN resection between the two procedures.
Zhang et al., 2016 [32]	VATS vs. thoracotomy	6247; VATS (n=2763), open (n=3484)	Systematic review and meta analysis	VATS harvested less total LN than open (95% CI −1.52 to −0.73, p<0.00001). N2 nodes were mainly poorly evaluated (95% CI −1.38 to −0.49, p<0.0001). Total LN stations evaluated were not different (95% CI −0.28 to 0.06, p=0.20).	Open lobectomy was superior to VATS in LN resection, especially N2 nodes.

Comparison of Long-Term Survival Rates

In 2014, a retrospective study conducted by Higuchi et al. sought to analyse the long-term outcomes of 160 Stage IA NSCLC patients, at Fukushima Red Cross Hospital, undergoing surgical resection by VATS (n=114) or open lobectomy (n=46) [28]. The five-year OS and five-year DFS for clinical Stage IA were 94.1% versus 81.8%, and 88% versus 72%, respectively, with a slight survival benefit being offered by VATS. Stephens et al. reported similar findings regarding five-year DFS somewhat favoring VATS. However, five-year OS was similar in propensity-matched analysis (VATS, 78% vs. open, 73%) [10].

In 2015, a study was conducted by Witte et al. including 100 patients with lung cancer who were either operated by VATS (n=56) or thoracotomy (n=44) [26]. The results showed that VATS conferred a significant five-year OS benefit when analysing all stages (86% vs. 69.9%) and Stage I separately (100% vs. 61.3%). The margin of benefit was narrower but still in favor of VATS when comparing five-year recurrence-free survival (58.5% vs. 48.6%). A fact to be noted here is that the thoracotomy group had more cases with a larger tumor diameter, which could have affected the long-term outcomes.

Zhong et al. also reported a slightly better long-term outcome with VATS supporting the previously mentioned studies (57.5% vs. 47.6% [five-year OS]; 45.2% vs. 35% [five-year DFS]) [24].

Hanna et al. conducted a propensity-matched retrospective study on 608 Stage I and II NSCLC patients treated surgically by VATS (n=196) or thoracotomy (n=412) [30]. They reported thoracotomy to confer nearly equivalent if not better survival benefit in terms of both five-year OS (VATS, 64% vs. open, 73%) and five-year DFS (VATS, 69.7% vs. open, 73%). This should be correlated with a previous finding stating that the efficacy of LN dissection was similar between the two groups. This study was more generalizable as it was larger than the previously mentioned ones regarding participants. Selection bias was also somewhat diminished by propensity matching of cohorts. Another point to mention is that the study accounted for Stages IB, II and IA, but no deductions can be made about the more advanced disease (Stage III or more).

To include a perspective of surgical efficacy of resection in advanced disease, we included a multi-institutional Chinese study conducted by Chen et al. that included 411 patients with locally advanced lung cancer (Stages IIA, IIB, IIIA) [25]. The results showed similar five-year OS (VATS, 55% vs. thoracotomy, 57.1%) and five-year DFS (VATS, 49% vs. thoracotomy, 42.2%) between the two procedures using propensity-matched analysis. A point worth noting was that a certain fraction of patients were converted from VATS to thoracotomy intraoperatively. When considering them in the open group, VATS conferred a better survival benefit. An important limitation of this study was that the database lacked information on neo-adjuvant therapy, which might have impacted advanced disease.

In 2014, Paul et al. compared the long-term outcome following VATS and thoracotomy in 6008 NSCLC patients from the SEER-Medicare database [27]. After propensity matching, the outcomes showed no difference in the long-term survival (three-year OS, VATS, 70.6% vs. thoracotomy, 68.1%; three-year DFS, VATS, 86.2% vs. thoracotomy, 85.4%). These results should be further interpreted, considering that the thoracotomy group had more patients with advanced-stage disease and larger tumors. However, compared to the previous studies, this study is most generalizable due to a significantly larger cohort size from a broad-spectrum US-based database. The same year, Liu et al. echoed findings similar to the ones previously mentioned that VATS resulted in an equivalent if not better long-term outcome [31]. This result has been somewhat consistent, except for one study by Hanna et al. stating otherwise.

Merritt et al., in their retrospective analysis of 129 NSCLC patients, also concluded that VATS provided equivalent survival benefit compared to thoracotomy (three-year OS, VATS, 89.9% vs. thoracotomy, 84.7%) [13]. However, this study was limited by its small sample size. Also, a point worth mentioning is that more patients in the open group received adjuvant chemotherapy, which should have tilted the benefit of thoracotomy. Still, the results showed otherwise. Lee and colleagues also did not report significantly different long-term survival rates between VATS and thoracotomy in their study [16]. They concluded that VATS was not inferior to thoracotomy in conferring a better oncological prognosis.

For the last two studies by Merritt et al. and Lee et al., although LN evaluation was better with thoracotomy in terms of the number of LNs and nodal stations dissected, this did not lead to the OS and DFS being in favor of open procedure.

In a consensus, our analysis showed that irrespective of LN evaluation status, VATS offers an equivalent if not slightly better long-term survival benefit in patients with NSCLC. The detailed descriptions of each study, including the year of publication, author name, number of patients, type of study, results, and authors' conclusions regarding the long-term survival rates (OS, DFS), are listed in Table 5.

**Table 5:** Synopsis of articles comparing VATS and thoracotomy in terms of long-term survival rates VATS, video-assisted thoracoscopic surgery; OS, overall survival; DFS, disease-free survival

Author and year of publication	Intervention studied	Number of patients	Type of study	Result	Conclusion
Stephens et al., 2014 [10]	VATS vs. open lobectomy	963; VATS (n=307), open lobectomy (n=656)	Retrospective cohort	OS at five years, unmatched group (n=963), VATS 78% vs. open 68%; propensity-matched analysis (n=600), VATS 78% vs. open 73%. Kaplan-Meier curves for disease-free survival favored VATS in unmatched analysis.	OS and DFS were similar between the two cohorts in propensity-matched analysis.
Zhong et al., 2013 [24]	VATS vs. open lobectomy	157; VATS (n=67), open lobectomy (n=90)	Retrospective cohort	Five-year OS, VATS 57.5% vs. open 47.6%; five-year DFS, VATS 45.2% vs. open 35%.	OS and DFS were not significantly different.
Chen et al., 2016 [25]	VATS vs. thoracotomy	411; VATS (n=250), thoracotomy (n=161)	Retrospective cohort	Five-year OS, VATS 55% vs. open 57.1%; five-year DFS, VATS 49.1% vs. open 42.2%). OS and DFS were similar between the two groups when comparing the same clinical stages: Stage IIa (56.4% vs. 66.8% [OS], 50% vs. 53% [DFS]), Stage IIb (62% vs. 72.2% [OS], 51.8% vs. 40% [DFS]), Stage IIIa (48.4% vs. 41.4% [OS], 44.2% vs. 30% [DFS]).	Five-year OS and DFS were similar between the two groups.
Witte et al., 2015[26]	VATS vs. thoracotomy	100; VATS (n=56), thoracotomy (n=44)	Retrospective cohort	Five-year OS (all stages), VATS 86% vs. open 69.9%; five-year OS (Stage I), VATS 100% vs. open 61.3%; five-year recurrence-free survival (all stages), VATS 58.5% vs. open 48.6%; five-year recurrence-free survival (Stage I), VATS 63.9% vs. open 75%.	VATS was conferred a notable survival benefit approach and was not inferior to open approaches.
Paul et al., 2014 [27]	VATS vs. thoracotomy	6008; VATS (n=1293), open (n=4715)	Retrospective cohort	VATS vs open unmatched cohort, three-year OS (71.2% vs. 63.8%); three-year DFS (86.5% vs. 77.6%); propensity-matched analysis, three-year OS (70.6% vs. 68.1%), three-year DFS (86.2% vs. 85.40%).	Propensity-matched groups showed similar results for OS and DFS. VATS is not inferior to thoracotomy in terms of outcome.
Higuchi et al., 2014[28]	VATS vs. open lobectomy	160; VATS (n=114), open lobectomy (n=46)	Retrospective cohort	VATS vs. open lobectomy, five-year OS clinical Stage IA (94.1% vs. 81.8%), five-year OS pathological Stage IA (94.8% vs. 96.2%), five-year DFS clinical Stage IA (88% vs. 77.1%), five-year DFS pathological Stage IA (91.5% vs. 93.8%).	OS and DFS were similar between VATS and open lobectomy.
Merritt et al., 2013 [13]	VATS vs. open lobectomy	129; VATS (n=60), open (n=69)	Retrospective cohort	Three-year OS (VATS 89.9% vs. open 84.7%); Kaplan-Meier survival curves were similar for the two.	Although more nodal sampling was achieved by open approach, the overall survival rates remained the same.
Lee et al., 2013[16]	VATS vs. thoracotomy	416; VATS (n=208), thoracotomy (n=208)	Retrospective cohort	VATS vs. thoracotomy, three-year OS (87.2% vs. 80.9%), five-year OS (74.9% vs. 76.6%), three-year DFS (78% vs. 74.7%), five-year DFS (60% vs. 70.3%).	VATS was not inferior to thoracotomy in terms of long-term outcomes.
Hanna et al., 2013 [30]	VATS vs. open lobectomy	608; VATS (n=196), open (n=412)	Retrospective cohort	Five-year OS (VATS 64% vs. open 73%), five-year DFS (VATS 69.7% vs. open 69.1%).	OS and DFS were not significantly different between the two cohorts.
Liu et al., 2014 [31]	VATS vs. thoracotomy	212; VATS (n=123), open (n=89)	Retrospective cohort	VATS vs. thoracotomy, three-year OS (79.2% vs. 72.6%), five-year OS (71.6% vs. 68%), three-year DFS (75.3% vs. 70.1%), five-year DFS (59% vs. 58.2%).	VATS and thoracotomy were similar in long-term outcomes.

Limitations

Our study had a few limitations, and hence the results should be interpreted keeping them in mind. Most importantly, there was only one RCT out of the 16 studies included in our systematic review. This might lessen the quality of evidence and raise concerns about the lack of prospective RCTs on the topic. Second, we included only English papers that might have led to language bias. Third, as most of our studies were observational, that calls for inherent selection bias as larger, more central tumors tend to be operated by open lobectomy by most surgeons. Also, we must take into account that a surgeon's preferred practice and expertise might also have led to selection bias. However, this aspect was somewhat lessened as most of the studies showed results after propensity matching of the cohorts. Fourth, we must account for information bias due to the non-availability and non-uniformity of certain data collected from various databases worldwide. Finally, the ideology and methodology of LN dissection or sampling and levels of expertise may vary between different surgeons, which may also have implications for the results.

## Conclusions

This systematic review was carried out to compare a minimally invasive VATS procedure with open thoracotomy in terms of their resection potential and long-term prognosis in patients with NSCLC to find out the surgical approach deemed more suitable ultimately. VATS is equivalent to thoracotomy in surgical resection of total LNs and LN stations. However, the data collected suggests that if only MLND is considered, a thoracotomy may offer a slightly better result. Also, nodal upstaging was more common with an open approach. On analysis of another aspect of long-term outcomes or survival rates, the evidence showed that VATS offered an equivalent if not slightly better result in overall survival and disease-free survival.

This review study will prove helpful in guiding practicing surgeons who are ambivalent regarding the optimal surgical technique. Irrespective of increased rates of nodal upstaging by thoracotomy, VATS should be considered a highly efficient alternative in both early and locally advanced NSCLC because of the marginally better survival benefit conferred. However, we found that very few RCTs have been conducted on the topic, and thus these findings should be ideally validated by a high-quality RCT.
